# COVID-19 in children and adolescents with neuroimmunological disorders

**DOI:** 10.1016/j.clinsp.2022.100142

**Published:** 2022-11-17

**Authors:** Ingrid Lacerda Pessoa, Renata Barbosa Paolilo, José Albino da Paz

**Affiliations:** Department of Neurology, Hospital das Clínicas da Faculdade de Medicina da Universidade de São Paulo (HCFMUSP), São Paulo, SP, Brazil

## Introduction

The disease associated to SARS-CoV-2 infection, COVID-19, posed a global public health challenge and impact. In Brazil, more than 600,000 deaths were reported until December 2021. The pediatric population was affected with approximately 2,500 deaths and 34,000 hospitalizations, leading to a 7% lethality rate among hospitalized children.^1^^,^^2^ Pediatric lethality in the US is about 14 times less common than in Brazil,^2^ which might be explained by a higher impact of pediatric COVID-19 in low-income countries.^3^

Especially in the first disease waves, epidemiologic studies proved COVID-19 is less severe in children than adults.^4^ Less than 5% of all pediatric cases are severe, and almost 16% of all pediatric cases are asymptomatic. Mild and moderate disease manifestations were reported in approximately 80% of pediatric patients.^5^, ^6^, ^7^ The most common disease manifestations are fever (50%), coughing (37%), and odynophagia (23%), while diarrhea, nasal obstruction, and dyspnea occur in a minority of cases.^7^^,^^8^ A Brazilian cohort evaluated 11,613 pediatric patients and concluded that 4,566 (40%) needed oxygen support; 1,167 (10%) needed invasive ventilation, and 886 (7.6%) patients died with a mean of six days from hospital admission.^9^

Multisystem Inflammatory Syndrome in Children (MISC) was also described in the early months of the pandemic. This condition is associated with COVID-19, and neurologic complications were reported.^10^, ^11^, ^12^

The described risk factors for disease severity are age (children under two years of age or adolescents over 12 years of age) and the diagnosis of chronic disorders, including some neurologic and immunocompromising conditions.^9^^,^^13^

Neurologic manifestations associated with COVID-19 were reported in40% of pediatric hospitalized patients from a multinational cohort. Symptoms comprised headache (20%), encephalopathy (16%), seizures (8%), encephalitis (1.3%), and stroke (0.9%).^14^ A study from the UK described the prevalence of neurologic complications in 3.8/100 pediatric hospitalized patients. The authors identified two different groups: neurologic complications associated with COVID-19 and neurologic complications associated with MISC. Encephalopathy was the most common manifestation (88%), especially in the MISC group. Almost half of the patients from the first group developed immune-mediated disorders such as Acute Disseminated Encephalomyelitis (ADEM), acute demyelinating disorders, and Guillain-Barré Syndrome (GBS).^15^ Other reported neurologic complications are disorders of the peripherical nervous system (15.8%), cranial neuropathies (9.7%), intracranial hypertension (4.6%), acute brain edema (2%), and cerebellar disorders (1%).^16^

Considering the increase of COVID-19 and its complications among children and adolescents, it is crucial to understand the disease's effects on particular groups of the pediatric population. The current editorial aims to discuss the impact of COVID-19 on children and adolescents with neuroimmunological disorders and under immunosuppressive therapy, as well as address the safety and efficacy of COVID-19 immunization in this specific group.

## COVID-19 in pediatric patients diagnosed with neuroimmunological disorders

Inflammatory conditions of the central and peripherical nervous systems such as Multiple Sclerosis (MS), a disease associated with myelin oligodendrocyte glycoprotein antibody ‒ MOG-IgG (MOGAD), ADEM, GBS, Myasthenia Gravis (MG), autoimmune encephalitis, and Opsoclonus Myoclonus ataxia Syndrome (OMS) are well-known to be triggered by several infections in some patients. This group of patients is generally treated with immunosuppressive drugs that might be associated with a lower cellular or humoral response. The combination of these two factors was the reason these patients were considered a higher fragility group in face of the COVID-19 pandemic.^17^^,^^18^

Unexpectedly, studies proved that both innate and adaptive immune responses are responsible for the inflammation and tissue damage seen in COVID-19. In this context, immunosuppression might not be harmful during SARS-CoV-2 infection. For now, literature reports that children under immunosuppressive therapies disclose similar disease manifestations and outcomes compared to other children. Thus, there is currently no recommendation to stop medications in suspected or confirmed COVID-19 scenarios.^19^^,^^20^

Studies evaluating the impact of COVID-19 in adult patients with neuroimmunological disorders showed that patients diagnosed with MG could disclose disease exacerbation and prolonged hospitalization.^21^^,^^22^ Although patients with MS did not disclose higher mortality from COVID-19,^23^ this infection has been shown to possibly trigger MS relapses.^24^ A study evaluating patients diagnosed with NMOSD suggested that rituximab treatment could be a risk factor for COVID-19 among these patients.^25^

There is little research evaluating the impact of COVID-19 on children with neuroimmunological disorders. In the most extensive study that enrolled 153 children with this diagnosis, 11% of patients had suspected or confirmed COVID-19. There was no difference in the frequency or severity of patients with or without immunosuppressive treatment, including rituximab. The identified risk factors were infected household contacts and low serum vitamin D levels.^26^

## COVID-19 vaccination in children and adolescents

Initially, the global immunization programs against COVID-19 did not include the pediatric population as a priority due to the understanding of the lower risk of complications in children and adolescents. With the advancement of the pandemic and the emergence of new variants of SARS-CoV-2 with a more significant potential for transmissibility, this agenda was intensely discussed and supported by national and international medical societies and public agencies in charge of vaccination.^2^^,^^27^^,^^28^

In order to achieve herd immunity, some countries started vaccinating pediatric groups in mid-2021. Preliminary studies have shown that vaccines against COVID-19 are safe and effective in children and adolescents, with current approval of 7 vaccines by the World Health Organization (WHO) for use in pediatrics, and more than 20 clinical trials are ongoing, including participants under the age of 18-years.^29^^,^^30^

In Brazil, adolescents over 12 years of age were initially included in the National Plan for the Operationalization of Vaccination against COVID-19 (PNO ‒ Plano Nacional de Operacionalização da Vacinação contra a COVID-19) in October 2021, and in January 2022, children from 5‒12 years of age were also included in the PNO.^31^ In July 2022, the National Health Surveillance Agency (ANVISA ‒ Agência Nacional de Vigilância Sanitária) approved an expansion of vaccination for children over 3 years of age.^32^

At the time of writing, two vaccines are approved for children and adolescents in Brazil: BNT162b2 (Pfizer/BioNTech) for children over 5 years of age, and CoronaVac (Sinovac) for children over 3 years of age. Both vaccines consist of a two-dose schedule for the general pediatric population, the first with an 8-week interval between the doses and the second with a 4-week interval between doses. Immunocompromised adolescents over 12 years of age should receive the Pfizer vaccine with a 3-dose schedule and a booster dose should be given 4 months after the third one.^31^^,^^33^

Vaccine authorization was based on randomized clinical trials. The use of the BNT162b2 vaccine in adolescents was evaluated in a placebo-controlled, phase 3 study that included 2260 participants between 12 and 15 years of age. Individuals using immunosuppressants were not included. A good safety profile similar to young adults was observed. Adverse effects were classified into mild or moderate, lasting one to two days. In general, systemic adverse events were reported more frequently after the second dose, and no severe vaccine-related adverse events were described.^34^ The same study proved efficacy in reaching the non-inferiority criteria for adolescents between 12 to 15 years of age compared to young adults. Compared to the placebo group, participants did not disclose COVID-19 seven days after the second dose, proving 100% efficacy.^34^ After a 6-months extension phase, participants maintained a high safety and efficacy profile (91.3%).^35^

The BNT162b2 vaccine was also evaluated in children from 5 to 12 years of age in a phase 2/3 trial. Most adverse effects were mild to moderate, and no participant presented with myocarditis or pericarditis. Vaccine efficacy was reported as 90.7% after a mean follow-up of 2.3-months.^36^

CoronaVac was evaluated in children from 3 to 17 years of age in a phase 1/2 randomized clinical trial. Up to 29% of participants reported mild and moderate adverse effects. Seroconversion of neutralizing antibodies was observed in 100% of subjects who received the 3.0 μg dose of the vaccine during phase 2. The trial concluded that this vaccine was well tolerated and responsible for humoral immune response in children and adolescents.^37^

## COVID-19 vaccination and neuroimmunological disorders

Despite the advances in vaccination in the pediatric age group, the immunization of children and adolescents with neuroimmunological diseases and under immunosuppressive therapies has not yet been widely studied. Although categorized as a priority in vaccination policies, this population has been excluded from most clinical trials of immunizers. One of the main reasons for this exclusion is that immunosuppressive drugs can hamper the effectiveness analysis of the agent once they interfere with humoral and cellular immune responses.^28^^,^^38^

About six clinical trials and observational studies are underway to evaluate the safety and immunogenicity of vaccines against COVID-19.^30^^,^^38^ To date, these data are scarce and clinical practice is often based on available information on other age groups or other vaccines that are already well-established in the vaccination schedule of the pediatric population using immunosuppressants.^39^

The Scientific Department of Neuroimmunology of the Brazilian Academy of Neurology (DCNI/ABN – Departamento Científico de Neuroimunologia da Academia Brasileira de Neurologia) and the Brazilian Committee for Treatment and Research in Multiple Sclerosis and Neuroimmunological diseases (BCTRIMS ‒ Comitê Brasileiro de Tratamento e Pesquisa em Esclerose Múltipla e Doenças Neuroimunológicas) recently published recommendations on general and COVID-19 vaccinations in adults living with the Central Nervous System (CNS) demyelinating disorders. The document concludes that the authorized COVID-19 vaccines are safe for immunosuppressed patients, and no modification of therapies is needed.^40^

An observational study including adults diagnosed with rare neuroimmunological disorders (NMOSD, MOGAD, and transverse myelitis) concluded that COVID-19 vaccines are safe and well-tolerated. Adverse events were similar to the general population and lower in participants under rituximab treatment (33%) than in other treatments (67%). 16% of patients reported the emergence or worsening of neurological symptoms, most of them self-limited sensory symptoms.^41^

Real-world evidence studies assessed vaccination safety in a group of patients with specific disorders. Eight of 56 patients with MG reported new neurologic symptoms after vaccination, and 37% required treatment with higher doses of steroids.^42^ MS patients did not present with increased disease relapses after Oxford/AstraZeneca or Pfizer vaccines.^43^^,^^44^

Regarding the efficacy of vaccines, immunosuppressive drugs seem to influence the immunogenicity of immunizers. Although COVID-19 vaccines generally reduce symptomatic infections in individuals under immunosuppressants, their efficacy is lower than that observed in the general population (70%).^45^ High-dose steroids and B cell depletion therapies were respectively associated with a 36 and 10-times reduction in humoral immune response compared to healthy controls.^46^

A prospective study evaluating 3,682 patients with rheumatologic disorders, 546 with MS, and 1,147 healthy controls after COVID-19 vaccination concluded that the seroconversion rate was similar in the groups, including patients under steroid treatment (89%‒100%). However, the patients under B cell depletion therapies had significantly lower seroconversion (43%).^47^ MS patients under natalizumab, teriflunomide, azathioprine, fingolimod, ocrelizumab, and rituximab treatment who received the Pfizer vaccine showed a lower humoral response compared to non-treated patients.^48^ Fingolimod was also associated with a lower cellular immune response.^49^

Medical societies and experts that commonly treat individuals with immune-mediated diseases recommend vaccination against COVID-19. The decision process must be shared with the patient and family. The best moment for vaccination should be individualized and, ideally, in a period of stability of the underlying disease. This group of patients should be informed about the possible reduction in vaccine effectiveness due to medications and encouraged to maintain preventive measures such as social distancing and hand hygiene. In addition, it is vital to reinforce the importance of vaccinating caregivers and household contacts as a protective measure.^17^^,^^40^^,^^50^

## Conclusion

Evaluation of COVID-19 impact on children with neuroimmunological diseases and using immunosuppressants is scarce. Despite the advancements in vaccination in the pediatric age group, the safety and efficacy of immunizations in children and adolescents living with neuroimmunological disorders have not yet been widely studied. More studies are needed to analyze the clinical manifestations and impact of COVID-19 in the pediatric population with neuroimmunological diseases.

## Authors’ contributions

Ingrid Lacerda Pessoa: Conceptualization, writing – original draft, review and editing.

Renata Barbosa Paolilo: Conceptualization, writing – review and editing.

José Albino da Paz: Conceptualization, validation, review and editing.

## Funding

This research did not receive any specific grant from funding agencies in the public, commercial, or not-for-profit sectors

## Conflicts of interest

The authors declare no conflicts of interest.

Ingrid Lacerda Pessoa: Reports no disclosure.

Renata Barbosa Paolilo: Reports grants from Roche and Merck and speaker honoraria from Novartis, outside the submitted work.

José Albino da Paz: reports no disclosure.
